# Metabolic checkpoints in glioblastomas: targets for new therapies and non-invasive detection

**DOI:** 10.3389/fonc.2024.1462424

**Published:** 2024-11-29

**Authors:** Wenhao Li, Zhihao Wang, Siliang Chen, Mingrong Zuo, Yufan Xiang, Yunbo Yuan, Yuze He, Shuxin Zhang, Yanhui Liu

**Affiliations:** ^1^ Department of Neurosurgery, West China Hospital, Sichuan University, Chengdu, China; ^2^ Department of Pediatric Neurosurgery, West China Second University Hospital, Chengdu, China; ^3^ Department of Critical Care Medicine, West China Hospital, Sichuan University, Chengdu, China

**Keywords:** glioblastoma, metabolic checkpoints, immunotherapy, multimodal MRI, liquid biopsy

## Abstract

Glioblastoma (GBM) is a highly malignant tumor of the central nervous system that remains intractable despite advancements in current tumor treatment modalities, including immunotherapy. In recent years, metabolic checkpoints (aberrant metabolic pathways underlying the immunosuppressive tumor microenvironment) have gained attention as promising therapeutic targets and sensitive biomarkers across various cancers. Here, we briefly review the existing understanding of tumor metabolic checkpoints and their implications in the biology and management of GBM. Additionally, we discuss techniques that could evaluate metabolic checkpoints of GBM non-invasively, thereby potentially facilitating neo-adjuvant treatment and dynamic surveillance.

## 1 Introduction

Glioblastoma (GBM) is the most common malignant glial tumor, accounting for nearly 50% of new brain malignant parenchymal tumors (1). Even with a combination of surgical resection, radiotherapy, temozolomide (TMZ) chemotherapy, and electric field therapy, the current median overall survival for GBM patients remains limited to 16-20 months (2). In recent years, immune checkpoint inhibitors (ICIs), including anti-CTLA-4 and anti-PD-1/PD-L1, have made breakthroughs in the treatment of solid tumors such as melanoma, lung cancer and kidney cancer (3–5). However, ICIs have failed to improve overall survival of GBM patients in Phase III trials (6, 7). In addition to ICIs, other promising immunotherapy strategies including tumor vaccines and adoptive cell therapies have shown limited progress in the treatment of GBM (6–9).

The resistance of GBMs to immunotherapies has been associated with the aberrant metabolic patterns. In GBMs, the glioma cells were observed to overexpress indoleamine 2, 3-dioxygenase (IDO) and tryptophan 2, 3-dioxygenase (TDO), which catabolize an excessive amount of tryptophan into kynurenine. Tryptophan is essential for effector T cell activation and maturation (10). Its depletion from the tumor microenvironment could result in cell cycle arrest and anergy of these cells. Additionally, kynurenine binds to the aryl hydrocarbon receptor (AHR) in the tumor-associated macrophages (TAMs) to facilitate expression of multiple immunosuppressors, e.g. ectonucleotidase CD39, which could reduce transcription of proinflammatory factors in CD8+ T cells through adenosine elevation in the GBM microenvironment (11). Similar to the notion of ‘immune checkpoint’ that regulates the execution of effector T cell cytotoxicity, Wang & Green defined ‘metabolic checkpoint’ to describe the impact of microenvironmental metabolites on immune cell function. With the growing application of immunotherapies in recent years, this concept has increasingly been adopted to describe the interconnection between the metabolic adaptations of tumors and their immunosuppressive microenvironment (10). Another metabolic checkpoint observed in GBM was the high levels of lactate produced in both the tumor cells and the TAMs due to their metabolic switch to aerobic glycolysis. By histone lactylation, excessive lactate enhances interleukin-10 (IL-10) expression, required for the suppression of T cell activity (12). These additional findings suggest potential diversity and intricacy in GBM metabolic checkpoints, compared to the relatively straightforward mechanism of immune checkpoints.

Despite growing recognition of the role of metabolic checkpoints in GBM, their complete landscape and clinical implications remain incompletely described due to limited current evidence. Here, we review current understanding of metabolic checkpoints in tumors and their potential links with metabolic adaptations in GBM. We also discuss recent evidence on the diversity and plasticity of tumor metabolic checkpoints, as well as their therapeutic implications for GBM. Finally, we highlight technical advancements in detection and surveillance of tumor metabolic checkpoints that may facilitate their future applications in GBM management.

## 2 Metabolic checkpoints in glioblastoma

### 2.1 Glucose metabolism and hypoxia

**Figure 1:**
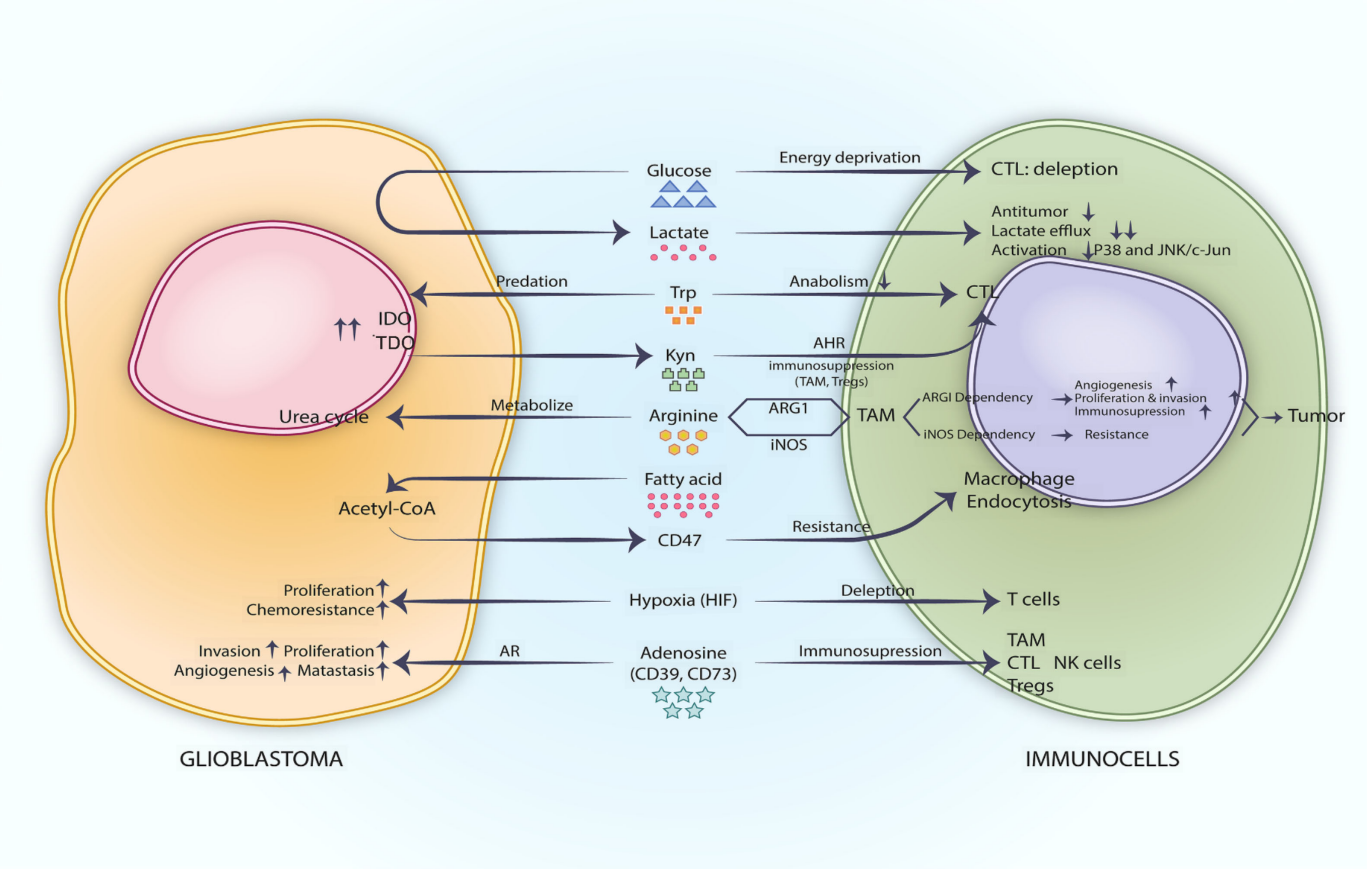
Metabolic checkpoints in the glioblastoma microenvironment.

Under aerobic conditions, cells typically metabolize glucose primarily through oxidative phosphorylation, generating substantial adenosine triphosphate (ATP) to meet cellular energy demands. However, even in the presence of oxygen, tumors utilize glucose through both oxidative phosphorylation and glycolysis, exhibiting a high rate of aerobic glycolysis. The phenomenon whereby cancer cells rely on glycolysis even in the presence of oxygen is termed the Warburg effect (13). In GBM, vigorous aerobic glycolysis generates substantial energy, resulting in the accumulation of lactate, a byproduct of this metabolic process, within the microenvironment. This accumulation modifies the lactate concentration and pH of the microenvironment (14). Karin et al. observed elevated lactate levels in the serum of 140 patients with various malignant tumors and found that prolonged exposure of T cells to high lactate concentrations impaired their proliferation (13). p38, JNK, and c-Jun are key molecules in the functional pathways of cytotoxic T lymphocytes (CTLs). Anna et al. found that lactate stimulation reduced the phosphorylation of these downstream molecules in CTLs and CTLs’ function was inhibited (15). In GBM, tumor cells seize glucose from the tumor microenvironment by virtue of vigorous aerobic glycolysis, which leads to depletion of CTL cells due to lack of energy, and the large amount of lactate produced by tumor cells in the microenvironment can further inhibit CTL antitumor immune function by obstructing CTL lactate efflux and p38 and JNK/c-Jun-mediated CTL activation, promoting M2-like polarization of microglia and other ways (13, 15, 16) (**Figure 1**). When a tumor grows rapidly, the supply of oxygen to the tumor cells from blood vessels becomes insufficient, resulting in a hypoxic tumor microenvironment (17). In glioblastoma, hypoxia is a critical feature of its microenvironment, aiding in the stabilization of hypoxia-inducible factors (HIFs), promoting tumor proliferation, and inducing T-cell depletion. In the tumor microenvironment, hypoxia can lead to the accumulation of adenosine, which exerts an inhibitory effect on antitumor responses by acting on immune cells (18). CD39 and CD73, which are involved in the conversion of ATP to adenosine, can act on glioblastoma stem cells and immune cells through adenosine receptors (AR), affecting the secretion of inflammatory mediators, the differentiation and proliferation of immune cells, and enhancing the stemness characteristics of cancer cells, thereby intervening in immunosuppressive effects and pro-tumor functions (18). (**Figure 1**) Lim et al. evaluated the therapeutic effects of an adenosine A2A receptor antagonist, AZD4635, and an immune checkpoint inhibitor, Durvalumab, in a phase I clinical trial involving patients with solid tumors (19). The outcomes of this trial indicate that AZD4635, either as a monotherapy or in combination with immune checkpoint inhibitors, holds therapeutic promise. However, further clinical trials are required to assess its efficacy (**Table 1**).

**Table 1:** Clinical trials related to tumor metabolism checkpoint inhibitors.

Trial	Phase	Drug	Target	Patient Population	Reference
NCT02048709	I	Navoximod (GDC-0919)	IDO	recurrent/advanced solid tumors	(Asha Nayak-Kapoor et al.2018) (120)
NA	I	AZD4635	Adenosine A2A Receptor	relapsed/refractory solid tumors	(Emerson A Lim et al.2022) (19)
NCT01386632	II	Dichloroacetate (DCA)	pyruvate dehydrogenase	unresected, locally advanced head and neck squamous cell carcinoma	(Steven F Powell et al.2022) (32)
NA	I	Telaglenastat	glutaminase	advanced or metastatic solid tumors	(James J Harding et al.2021) (33)
NA	II	Indoximod	IDO	advanced melanoma	(Yousef Zakharia et al.2021) (121)
NCT02559492	I	Epacadostat	IDO	advanced solid tumors	(Aung Naing et al.2022) (122)

### 2.2 Amino acid metabolism

IDO and TDO are two crucial enzymes involved in the metabolism of tryptophan, serving as representative metabolic checkpoints in amino acid metabolism (20, 21). In GBMs, glioma cells are found to express elevated levels of IDO and TDO, which catabolize excessive amounts of tryptophan into kynurenine. Tryptophan is essential for the activation and maturation of effector T cells (10). Hanihara et al. demonstrated that the downregulation of IDO in glioma cells increased the survival time of tumor-bearing animals in experimental models (21). They observed that, following the downregulation of IDO in tumor cells, the percentage of T cells in the spleens of mice increased compared to the non-downregulated control group. However, in nude mice, downregulation of IDO did not result in improved survival. Consequently, IDO may influence tumor dynamics by modulating T cells. The downregulation of IDO resulted in the accumulation of tryptophan and a concomitant decrease in kynurenine levels. Tumor cells possess a robust capability to metabolize tryptophan, leading to the accumulation of their metabolic byproducts in the microenvironment (21). The depletion of tryptophan induces T cells to downregulate their anabolic pathways for tryptophan synthesis (21). TDO is highly expressed in human glioma cells, which aids in understanding the impact of tryptophan catabolism on immune cells (20). Opitz et al. demonstrated that the downregulation of IDO and TDO indicated that the tryptophan metabolite Kyn is primarily influenced by TDO in glioma cells (20). They observed that knocking down TDO affects T cell proliferation, whereas the addition of kynurenine (Kyn) restores this proliferation. Kyn serves as an agonist of the AHR. In experimental models, the absence of AHR diminished the tumor-promoting effects of TDO. This mechanism facilitates CTL immunosuppression by tumor-associated macrophages (TAM) and regulatory T cells (Tregs) via the binding of Kyn to the AHR (20, 22). IDO inhibitors can also be combined with other pharmacological agents to enhance their effects; for instance, IDO inhibitors have been shown to enhance the anti-tumor effects of TMZ in a mouse glioma model (21) (**Figure 1**).

Analogous to tryptophan, arginine also functions as a metabolic checkpoint. TAMs constitute a significant component of the glioma microenvironment, and their polarization state is linked to either tumor promotion or anti-tumor activity. Based on the distinct patterns of arginine metabolism exhibited by TAMs, they can be classified into Arginase 1 (ARG1)-dependent TAMs and cytokine-inducible nitric oxide synthase (iNOS) TAMs. The former exerts pro-tumor effects by promoting tumor invasion, migration, and immunosuppression through factors such as vascular endothelial growth factor (VEGF), signal transducer and activator of transcription 3 (STAT3), transforming growth factor-β (TGF-β), and interleukin-10 (IL-10). The latter exerts anti-tumor effects by secreting chemokines, tumor necrosis factor-α (TNF-α), interleukin 1 beta (IL-1β), nitric oxide (NO), and reactive oxygen species (ROS) (17) (**Figure 1**). Comprehensive research aimed at identifying metabolic checkpoint targets related to arginine metabolism may enhance the anti-tumor effects of immune cells.

### 2.3 Lipid metabolism

Lipid metabolism represents another critical energy metabolic pathway in the human body, generating substantial energy through β-oxidation to support various physiological functions. In tumor cells, fatty acid metabolism not only provides energy, promoting cell growth and proliferation, but also influences the tumor’s invasive potential (23). CD36 is a membrane glycoprotein located in the cell membrane that binds to extracellular fatty acids, participating in their transport and metabolic regulation (24, 25). A study on oral cancer revealed that tumors are characterized by elevated expression of lipid metabolism genes and the fatty acid receptor CD36. A high-fat diet has been shown to promote metastasis, whereas blocking CD36 can inhibit this process (26). In glioblastoma, lipid droplets accumulate in the pathological tissues of patients, and lipid metabolism enhances glucose metabolism and tumor proliferation. Inhibition of lipid metabolism results in a decrease in the tumor’s proliferative capacity (27). Tregs are immunosuppressive cells, and the upregulation of CD36 on intratumoral Tregs contributes to their function. Genetic ablation of CD36 reduces the presence of Tregs within the tumor and enhances the anti-tumor activity of other immune cells (28). Analogous to Tregs, myeloid-derived suppressor cells (MDSCs) can suppress immune cell function and promote tumor growth. As a heterogeneous population of cells, MDSCs are associated with cancer-related expansion. MDSCs can suppress T cell function, promote the expansion of Tregs, and facilitate tumor proliferation and migration (29, 30) In mouse tumor models, lipid metabolism is enhanced in tumor-infiltrating MDSCs. Inhibiting fatty acid oxidation can diminish the immunosuppressive functions of MDSCs and slow down tumor growth (30).

In the context of gliomas, metabolic checkpoints associated with lipid metabolism merit further investigation. The aberrantly active fatty acid oxidation (FAO) reaction in GBM can generate substantial quantities of acetyl Co-A to increase CD47 expression, which facilitates immune evasion through CD47 resistance to endocytosis and interference with FAO by etomoxir restores the endocytosis response of macrophages to tumors and also inhibits FAO-dependent MDSC and Tregs (31) (**Figure 1**).

## 3 Heterogeneity and plasticity of metabolic checkpoints

**Figure 2:**
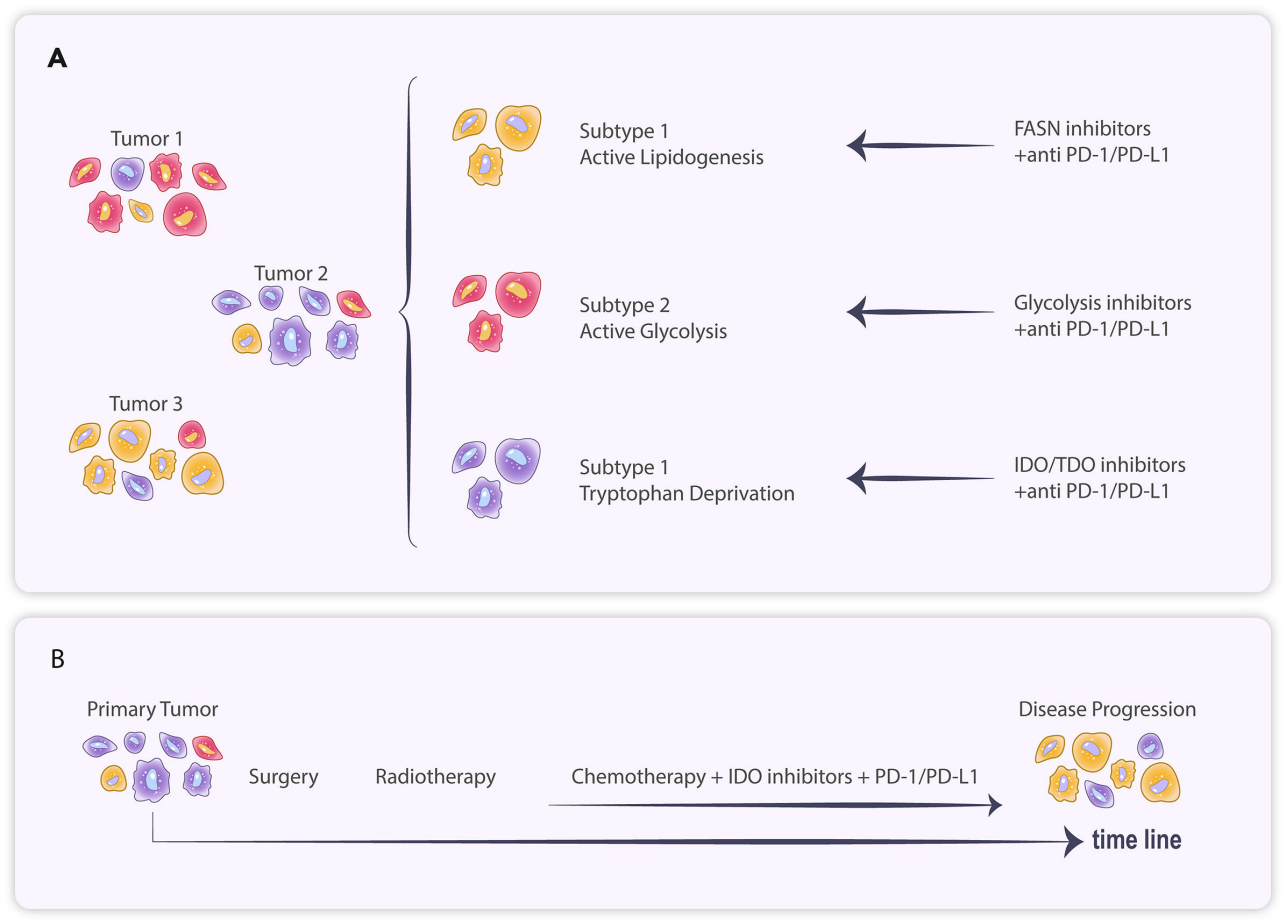
**(A)** Tumor cells of different subtypes exhibit distinct metabolic patterns and respond variably to treatment. **(B)** The primary tumor treated with systemic therapy resulted in branching evolution, ultimately leading to disease progression.

The protective effect of metabolic checkpoints on tumors suggests that their inhibitors have significant potential to enhance the efficacy of immunotherapy. Therefore, researchers are tirelessly exploring therapeutic targets related to metabolic checkpoints. There are also clinical trials of investigational drugs targeting of pyruvate dehydrogenase and glutaminase (32, 33) (**Table 1**). However, preliminary results from some clinical trials currently indicate variability in patient responses to metabolic checkpoint inhibitors. In two trials in uroepithelial carcinoma, the IDO1 inhibitor Epacadostat combined with pablizumab demonstrated a higher objective remission rate (ORR) compared to the pablizumab group, but no benefit was observed from combining Epacadostat in trials in other tumors (34). Notably, IDO1 expression exhibits considerable variability across different tumors, with some immunohistochemical studies suggesting IDO1 positivity rates of 94% in uroepithelial carcinoma, 57-66% in ovarian cancer, 37-46% in breast cancer, 44-81% in renal cancer and 8% in GBM (34). Therefore, it has been suggested that the failure of Epacadostat to perform as expected in clinical studies may stem from the variability in IDO1 expression among patients, and that these studies often fail to screen for potentially sensitive patients based on IDO1 expression levels at enrollment (34) (**Figure 2**).

Glutamine-related metabolic reprogramming in tumor cells enhances the survival of cancer cells (35). Glutaminase is a key enzyme in glutamine metabolism, and it serves as a target for anti-tumor therapy (36–39). IDH mutations in gliomas influence the glutamate synthesis pathway in tumor cells, resulting in an increased conversion of glutamine to glutamate in IDH mutant gliomas (40). Moreover, the levels of glutamine metabolism in liver tumors vary according to the induced gene, while notable differences exist between liver and lung cancers induced by the same gene (41). Thus, variations in the efficacy of the same metabolic checkpoint therapy may occur across different molecular subtypes of gliomas. Even when identical genes are involved, the impact of tissue heterogeneity on metabolic checkpoint therapy may be significant.

Due to the diversity of tumor metabolic checkpoints, studying the expression profiles of tumor metabolites and related pathway genes at the histological level is more conducive to identifying the key metabolic checkpoints involved. We can observe the heterogeneity of the tumor’s metabolic profile and the phenomenon of subgrouping in breast cancer (42). Shao et al. have identified three Metabolic-Pathway-Based Subtypes (MPS) in triple-negative breast cancer (TNBC). The MPS1 subtype is notably active in the lipid metabolism pathway, while the MPS2 subtype is significantly active in the glycolytic pathway. Drug trials targeting both metabolic profiles demonstrated that the fatty acid synthase (FASN) inhibitor C75 significantly inhibited the growth of MPS1 TNBC cell lines and patient-derived organoids compared to MPS2 and MPS3, whereas the glycolysis inhibitor was more effective against MPS2 TNBC. In a tumor-bearing mouse model, the combination of the glycolysis inhibitor FX-11 and PD-1 monoclonal antibody significantly increased the infiltration of CD8+ T cells and NK cells in MPS2 tumors, effectively slowing their growth, whereas no significant effects were observed in MPS1 and MPS3 tumors (43) (**Figure 2**).

Heterogeneity and clustering of tumor metabolic characteristics are not only evident in TNBC but also in various tumors, including lung cancer, gastric cancer, and glioma (44–48). One study categorized gastric cancer into four subtypes with distinct metabolic profiles, with each subtype’s prognosis varying across treatments (49). Diffuse low-grade gliomas were classified into three distinct metabolic subtypes (M1, M2, and M3) through consensus clustering, differing in immune infiltration, molecular characteristics, and prognosis (50). Metabolomic assays of GBM cell lines demonstrated that these lines could be categorized into multiple groups with significantly different metabolic profiles (51). Chinnaiyan et al. found significant metabolomic heterogeneity in GBM (52). Furthermore, GBM can be differentiated into AD-H and AD-L types through hierarchical clustering of relative levels of adenosine-related metabolites; additionally, immunocell infiltration analysis indicated that M2 macrophage infiltration was significantly higher in AD-H than in AD-L (53). This body of evidence suggests that the expression levels of metabolic checkpoints vary significantly among GBMs, indicating that a holistic approach to determining the metabolic profile classification through histological testing may be essential for effective intervention (**Figure 2**).

In addition to variations in metabolic checkpoint expression among patients, the compensatory and adaptive evolution of metabolic pathways may also contribute to the eventual tolerance of tumors to metabolic checkpoint inhibitors. Kunle et al. found that ovarian cancer patients treated with the IDO1 inhibitor Epacadostat experienced a shift in internal tumor tryptophan metabolism toward the NAD+ synthesis pathway, which also affected T cell infiltration and anti-tumor immune function. In contrast, the addition of A2a and A2b purinoceptor antagonists to block their binding to NAD+ alleviated immunosuppression (54). In GBM, although no studies have examined changes in tumor dynamics following treatment with metabolic checkpoint inhibitors, evidence suggests that neoantigen-containing tumor clones in GBM (Responders) that are sensitive to immune checkpoint inhibitor treatment may undergo branched evolutionary elimination, leading to eventual progression (55). Therefore, this form of tumor adaptive evolution may also account for the eventual failure of treatments targeting immunosuppression (**Figure 2**).

## 4 Non-invasive tests for predicting metabolic checkpoints

### 4.1 Multimodal MRI

Given that GBM is located intracranially, it is essential to capture the key metabolic checkpoints of GBM in a non-invasive and dynamic manner to more accurately match patients with more effective or sensitive drugs, considering the heterogeneity and plasticity of tumor metabolic checkpoints.

**Figure 3:**
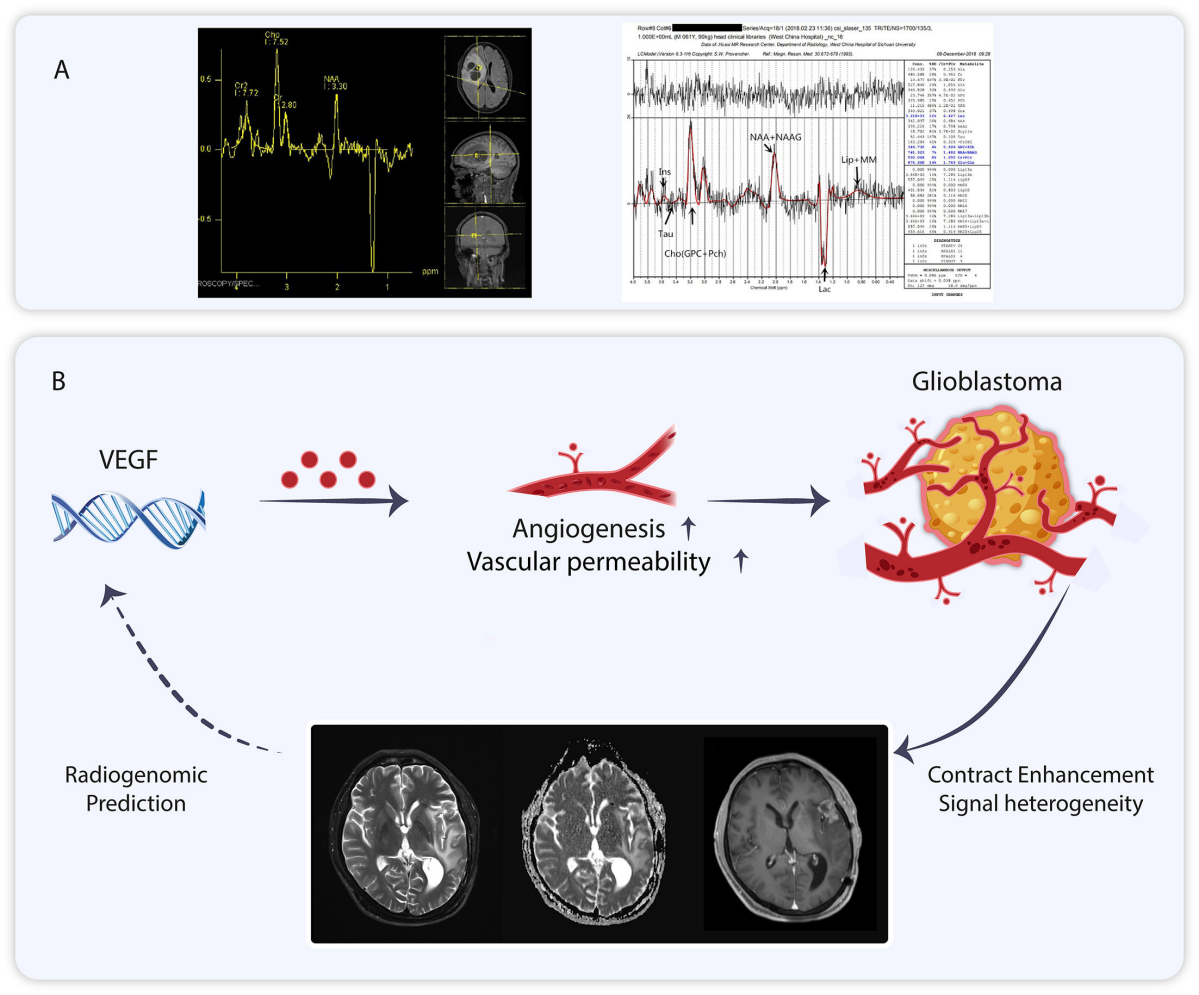
**(A)** Nuclear magnetic spectroscopy imaging of glioblastoma. **(B)** The role of vascular endothelial growth factor for glioblastoma can be visualized using Magnetic Resonance Imaging, and MRI analysis can be employed to predict vascular endothelial growth factor expression.

Multimodal magnetic resonance imaging(MMRI) is a combination of various MRI imaging modalities (56). The individual imaging modalities can complement each other (57). MMRI has been utilized in various diseases, including osteosarcoma, Parkinson’s disease, concussion, and glioma (58–61). MMRI is increasingly employed in the management of gliomas (62). Proton magnetic resonance spectroscopy (^1^H-MRS) is part of MMRI, and the application of magnetic resonance spectroscopy (MRS) to measure brain metabolites dates back 40 years (63, 64). ^1^H-MRS can be directly utilized to estimate the relative levels of various metabolites, such as glutamine, lactate, and lipids, in the human GBM region, providing metabolic characteristics of tumors at different spatial and temporal points (65–67). This technique can be adapted to fulfill the need for non-invasive assessment of metabolic checkpoints. In addition to human GBM, there is concordance between ^1^H-MRS of living tumors and isolated tissues in a rat glioma model (68). In studies of anti-angiogenic drug response, the sensitive population (long-term survivors) demonstrated a consistently lower lactate/N-acetylaspartate ratio (Lac/NAA) compared to baseline during continuous ^1^H-MRS follow-up, whereas the non-sensitive population exhibited a gradual increase in Lac/NAA (69). Additionally, phosphorus magnetic resonance spectroscopy (^31^P-MRS) aids in detecting cellular metabolites, and metabolite concentration ratios have been utilized in glioblastoma studies (70, 71). In one study, ^31^P-MRS combined with ^1^H-MRS was employed to detect changes in the biology of glioblastoma following treatment with bevacizumab (72). ^31^P-MRS can also identify various brain tumors due to differences in metabolite concentration ratios among different tumors (73, 74). Alongside ^1^H-MRS and ^31^P-MRS, deuterium MRS and carbon-13 MRS are also employed in tumor metabolism detection (75, 76) (**Figure 3**).

In addition to the aforementioned MRI spectroscopy, T1/T1 enhancement, T2, fluid-attenuated inversion recovery (FLAIR), and diffusion-weighted imaging (DWI) included in multimodal MRI enable the extraction of over 1,600 statistics that describe the signal morphology and texture of tumor images, collectively referred to as radiomics features. Radiomics is a sophisticated imaging analysis technique that can be integrated with other histological data for clinical applications (77). Radiomics has demonstrated significant potential in tumor research (78). Its value has been demonstrated in breast, ovarian, liposarcoma and glioblastomas (79–82). Gene functions influence the imaging characteristics of tumors. Zhang et al. found that contrast enhancement (CE) in T1-weighted sequences is associated with genes related to blood-brain barrier (BBB) function (83). Angiogenesis is a critical feature of GBM, and vascular endothelial growth factor (VEGF) plays a key role in regulating this process. VEGF is a significant target in glioblastoma, and its evaluation holds clinical importance (84, 85). MRI can predict VEGF expression in GBM through a non-invasive modality based on radiomics (85–87) (**Figure 3**).

The application of artificial intelligence in processing medical data is increasingly being adopted by researchers. Machine learning (ML) enables the utilization of known data to make predictions about unknown data (88). Deep learning, a branch of machine learning, is based on the study of neural networks and is classified into supervised, unsupervised, and semi-supervised learning (89). The integration of machine learning with imaging information can assist in the diagnosis, treatment, prognosis, and staging of diseases (90, 91).

In a previous study, we identified numerous imaging histological features in multimodal MRI of GBM that were highly correlated with gene expression in tumor tissues using automated machine learning process (92). This finding aligns with the results of numerous prior studies (93). For instance, by comparing GBM imaging and transcriptomic features, Beig et al. found that a set of imaging features with prognostic value were significantly associated with cell differentiation, adhesion, and angiogenesis (81). Li et al. identified 13 prognostic imaging features in GBM that were classified by WGCNA analysis into four categories of imaging phenotypes significantly associated with gene expression in pathways related to immunity (T cell activation, etc.), tumor proliferation (cell adhesion, etc.), therapeutic response (UV response, etc.), and cellular function (mitotic spindle, etc.) (94). These findings robustly demonstrate the feasibility of non-invasive analysis of gene expression through the correlation of imaging and transcriptomics.

Considering the aforementioned advantages, it can be concluded that multimodal MRI holds significant potential for predicting the expression of key metabolic checkpoints in GBM.

### 4.2 Liquid biopsy

Liquid biopsy has recently emerged as a non-invasive technique for detecting tumor biomarkers (95, 96). The temporal and spatial heterogeneity of tumors is an intrinsic property that limits the efficacy of traditional tissue biopsies (97). Traditional tissue biopsy remains the gold standard for pathological diagnosis; however, it is associated with the disadvantages of invasiveness and inconsistent sampling (98). Liquid biopsies offer the advantages of being non-invasive and providing a more convenient method for the continuous monitoring of tumor outcomes (99–101). Circulating tumor cells (CTCs) and circulating tumor DNA (ctDNA) are significant biomarkers in liquid biopsies (102). Circulating tumor DNA primarily derives from the death of tumor cells (102). In lung cancer, biomarkers obtained from liquid biopsies have been utilized to assess the therapeutic efficacy of immunotherapy (103). In the context of immunotherapy, results from clinical trials such as B-FAST and CheckMate 848 indicate that the tumor mutational load of circulating tumor DNA in plasma may suggest potential therapeutic benefits from immune checkpoint inhibitors (104, 105). In glioblastoma, cerebrospinal fluid (CSF) is considered more valuable than other body fluids; however, additional body fluids can also serve as fluid biopsy specimens for monitoring glioblastoma, with cell-free nucleic acids, extracellular vesicles (EVs), and CTCs being notable biomarkers (106, 107). Nevertheless, there are currently no studies demonstrating the presence of metabolic checkpoint-associated liquid biopsy markers.

**Figure 4:**
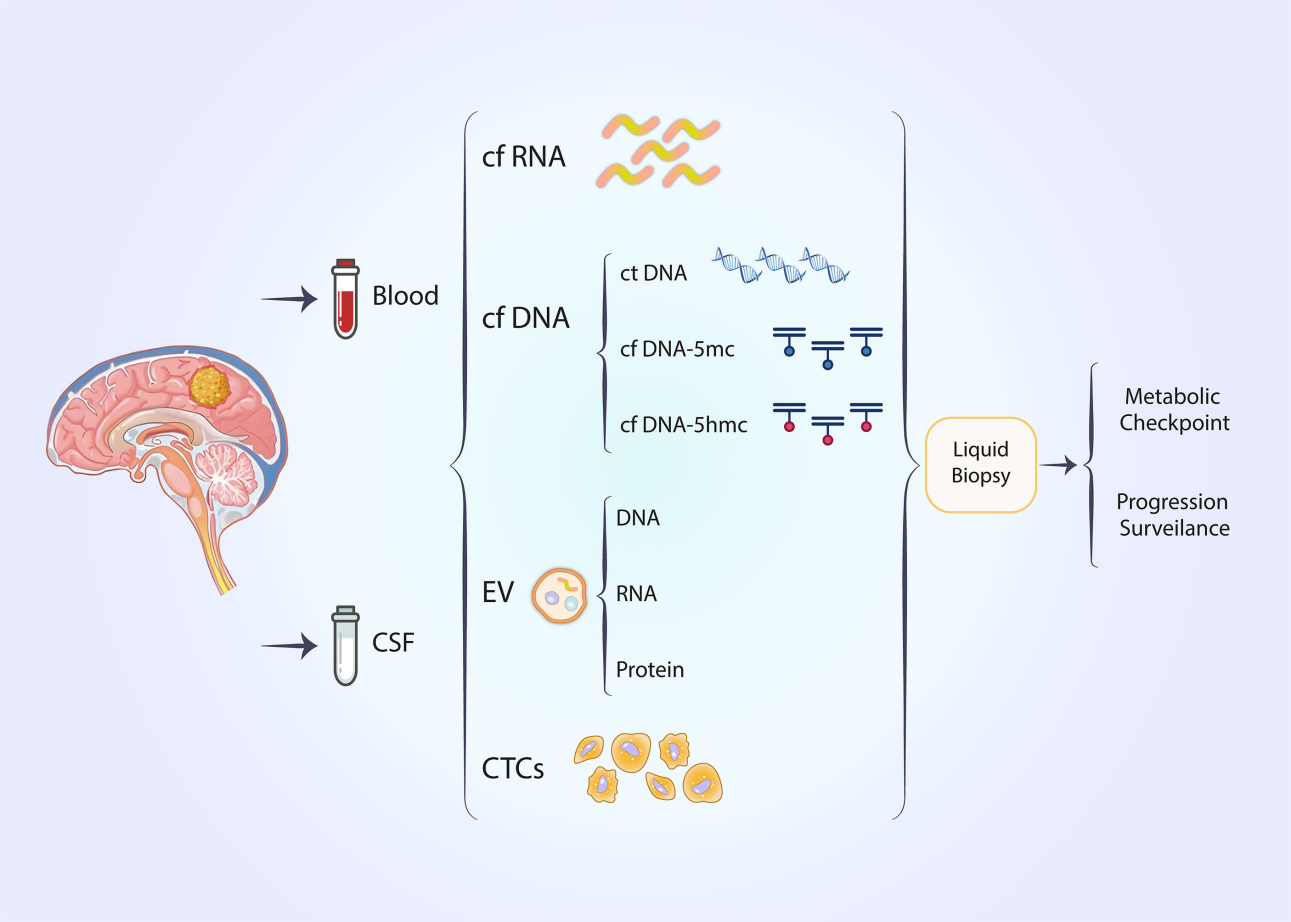
Noninvasive liquid biopsy techniques can be employed to predict metabolic checkpoints and progression surveillance in glioblastoma.

Epigenetic alterations may serve as promising liquid biopsy markers that could assist in the diagnosis and treatment of cancer (108, 109). DNA methylation is extensively studied in the context of epigenetic modifications (108). DNA methylation in circulating tumor cells is closely linked to cancer occurrence, and the frequency of methylation in gene promoter regions influences gene expression, thereby affecting cancer development (110). DNA methylation can be detected in the body fluids of patients with various solid tumors (95, 111–114) (**Figure 4**).

We achieved high-throughput detection of cytosine 5-hydroxymethylation (5hmc) in circulating free DNA (cfDNA) from glioma patients using a UDP-N3-glucose-based click chemistry combined with immunoprecipitation method (115). In contrast to the mutation or methylation of cytosine (5mc) in free DNA detected by most liquid biopsy methods,5hmc serves as a stable intermediate in the demethylation of methylated cytosines, thereby reflecting an open chromatin state (116). Specific enzymes mediate the conversion of 5mc to 5hmc (117). 5hmc in genes exhibits a positive correlation with gene expression, enabling this class of epimodification signals to more directly reflect the transcriptomic profile of tumors (116, 118). In esophageal cancer, 5hmc in cfDNA can serve as a biomarker for tumor detection (119) (**Figure 4**).

Utilizing the aforementioned 5hmc assay, we established a preliminary cfDNA hydroxymethylome dataset for a cohort of glioma patients and compared it with the glioma transcriptome, confirming that the level of gene 5hmc modification in plasma cfDNA of glioma patients closely correlates with its expression level in tumor tissue (115).

## 5 Future perspectives

Glioblastoma is a malignant tumor of the central nervous system, with surgery remaining the primary treatment modality. Due to its inherent heterogeneity, the obstruction of the blood-brain barrier, and the immunosuppressive characteristics of the tumor microenvironment, immunotherapy is less effective. Therefore, there is a pressing need to develop new therapeutic modalities for glioblastoma. Metabolic checkpoints offer a novel direction for the diagnosis and treatment of glioma, serving not only for diagnostic staging of glioblastoma but also for the identification of new therapeutic targets. Non-invasive analytical methods can aid in analyzing the metabolic checkpoints of glioblastoma, with radiomics and liquid biopsy offering unique advantages for diagnosis and treatment. However, further research on metabolic checkpoints in glioblastoma faces several limitations and challenges. Research on metabolic checkpoint targets in glioblastoma remains insufficient, and drugs targeting these specific sites are underdeveloped, necessitating further exploration. Given the high heterogeneity of glioblastoma, studies on metabolic checkpoints require more detailed classification. The integration of artificial intelligence and radiomics requires additional data support and advancements in AI technology. Non-invasive techniques for predicting metabolic checkpoints require further refinement.

In conclusion, glioblastoma metabolic checkpoints represent a promising area of research, necessitating further in-depth studies. The combination of liquid biopsy techniques and radiomics warrants further exploration.
